# An Investigation into the Appropriateness of Car-Following Models in Assessing Autonomous Vehicles

**DOI:** 10.3390/s21217131

**Published:** 2021-10-27

**Authors:** Akito Higatani, Wafaa Saleh

**Affiliations:** 1 Hanshin Expressway Co., Ltd., 7-15-26, Osaka 553-0003, Japan; higatani-akito@hanshin-exp.co.jp; 2 College of Engineering, Princess Nourah Bint Abdulrahman University, Riyadh 84428, Saudi Arabia; 3 Transport Engineering, Transport Research Institute, Edinburgh Napier University, 10 Colinton Road, Edinburgh EH10 5DT, UK

**Keywords:** autonomous vehicles, car-following models, Helly models, ITS

## Abstract

The dramatic progress of Intelligent Transportation Systems (ITS) has made autodriving technology extensively emphasised. Various models have been developed for the aim of modelling the behaviour of autonomous vehicles and their impacts on traffic, although there is still a lot to be researched about the technology. There are three main features that need to be represented in any car-following model to enable it to model autonomous vehicles: desired time gap, collision avoidance system and sensor detection range. Most available car-following models satisfy the first feature, most of the available car-following models do not satisfy the second feature and only few models satisfy the third feature. Therefore, conclusions from such models must be taken cautiously. Any of these models could be considered for updating to include a collision avoidance-system module, in order to be able to model autonomous vehicles. The Helly model is car-following model that has a simple structure and is sometimes used as the controller for Autonomous Vehicles (AV), but it does not have a collision avoidance concept. In this paper, the Helly model, which is a very commonly used classic car-following model is assessed and examined for possible update for the purpose of using it to model autonomous vehicles more efficiently. This involves assessing the parameters of the model and investigating the possible update of the model to include a collision avoidance-system module. There are two procedures that have been investigated in this paper to assess the Helly model to allow for a more realistic modelling of autonomous vehicles. The first technique is to investigate and assess the values of the parameters of the model. The second procedure is to modify the formula of that model to include a collision avoidance system. The results show that the performance of the modified full-range Auto Cruising Control (FACC) Helly model is superior to the other models in almost all situations and for almost all time-gap settings. Only the Alexandros E. Papacharalampous’s Model (A.E.P.) controller seems to perform slightly better than the (FACC) Helly model. Therefore, it is reasonable to suggest that the (FACC) Helly model be recommended as the most accurate model to use to represent autonomous vehicles in microsimulations, and that it should be further investigated.

## 1. Introduction 

The significant increase in technological advances and progress in Intelligent Transportation Systems (ITS) have enabled autodriving and Autonomous Vehicles (AV) technology and their related research to become possible. There are six stages of automation, where full automation, level five, is the final stage, and zero automation is level zero. Full automation means a driverless vehicle. If an autonomous vehicle is equal to a driverless vehicle, then partial autonomous vehicles (say level two or three) are already being sold in the market. There are some core technologies related to a driverless vehicle, such as collision avoidance system, auto steering technology, lane changing technology, intelligent parking systems, communication technology and sensor technology. Road-to-vehicle connecting technology such as Intelligent Speed Adaptation (ISA) is implicitly included in the communication technology. Furthermore, platoon technology may be also utilised as a core technology in the field of freight transport.

Many studies and researchers have investigated issues related to numerous aspects of autonomous vehicles. These include social and technological issues, and prediction of the market. Various models have been developed to model Autonomous Vehicles (AV), yet most of them are still using more or less the same principles of the models that have been developed for manually driven vehicles and driving behaviour. Therefore, conclusions from such models have to be taken cautiously. There is a great deal still unknown about this technology and its impacts on traffic behaviour and transportation systems. Car-following models are typically used as methods to investigate driving behaviour and the impact of the behaviour of the *lead* vehicle on the behaviour of the *following* vehicle. Various car-following models have been developed since the 1950’s to represent driver’s car-following behaviour such as the Gipps Model (GM), Collision Avoidance Model (CA model), Helly model, Optimal Velocity Model (OVM), Intelligent Driver Model (IDM) and Intelligent Driver Model+ (IDM+). Most of these models have also been used to model the AV behaviour, with or without modifications. 

The three main features of autonomous vehicles that are considered in this research are: (1) Auto Cruise Control technology. This is by far the most important technology in autonomous, driverless vehicles. Auto Cruise Control (ACC) technology and its full-range Auto Cruise Control (FACC) have been around for the past 60 years, at least. ACC is mainly a technology which adjusts the gap and sustains the desired gap using sensors. This means that the vehicle is able to maintain constant distance gap as well as constant speed, which results in reducing chances of interrupting flowing traffic; (2) The second feature of an AV is the collision avoidance system. Since all autonomous vehicles, in reality, will have this feature, autonomous vehicle controllers and models should have such a function. Any models that are used to represent AV appropriately should be verified regarding whether they have an appropriate braking function; and (3) a sensor detection range. A precise and fast sensor detection system is vital for autonomous vehicles and autodriving. This is important to be able to identify vehicles’ shapes, distances, speeds and movements. 

To conclude, for any model to be appropriately used to model behaviour of AV and their behaviour, it needs to satisfy the three requirements: the desired time gap, collision avoidance system and sensor detection range. Some models have limitations in representing varying desired gaps that depend on vehicle speed. For example, the GM model does not have a term for desired time gap or desired gap. Therefore, it cannot represent AV behaviour and it will have to be adjusted with the right parameters that depend on the speed. The four car-following models and controllers that will be investigated in this study all have a desired time-gap feature. These are the Helly model, IDM, IDM+ and Alexandros E. Papacharalampous’s Model (A.E.P.) controller. 

This study reports on an investigation of the appropriateness of car-following models in replicating the behaviour of autonomous vehicles. Data from vehicles that are equipped with full-range ACC (FACC) and are already commercially available in the market were obtained and utilised in the assessment. The Helly model, which is a very frequently used car-following model, is assessed and examined for appropriateness for update for the purpose of using it to model autonomous vehicles more efficiently. This involved assessing the parameters of the model and investigating a possible update to include a collision avoidance system component. An enhanced car-following model, Helly (FACC), is proposed, assessed and validated. The validation included a comparative analysis of models’ performance in two paradigms that are taken from the Japanese collision avoidance-system standards. These are: a paradigm that states that “when the following vehicle approaches at 50 km/h to a stationary vehicle, the following vehicle should not collide with the stationary vehicle or the following vehicle’s speed should be less than 20 km/h if a collision occurs”.

The paper is structured as follows. Section 2 presents previous related work. Discussions of car-following models and their appropriateness for modelling AV are also presented. Section 3 is devoted to the investigation of the appropriateness of the Helly model to represent AV. In this Section, the parameters of the model are tested and examined using case scenarios. In addition, modifying the model formulation as well as validation of the results are also presented in Section 3. Section 4 is devoted to the conclusions and further research recommendations.

## 2. Previous Related Work 

Microsimulation modelling and car-following models are typically used as methods to investigate the impact of autonomous vehicles’ operation and monitoring [1,2,3,4,5,6,7,8]. Various car-following models have been developed since the 1950’s to represent driver’s car-following behaviour such as the Gipps Model (GM) [4], collision avoidance model (CA model) [4], Helly model [5], Optimal Velocity Model (OVM) [6], Intelligent Driver Model (IDM) [7], and IDM+ [8]. 

Research on autonomous vehicles using microsimulation can be divided into two types. One is research using microsimulation with existing car-following models such as IDM and IDM+ [9]. Martin Treiber et al. [7] reproduced driving behaviour of autonomous vehicles using IDM and investigated the impact of autonomous vehicles on traffic flow [9]. Interestingly, they reported in the paper that human and automated driving behaviours are fundamentally different in terms of reaction time and driving strategies. The IDM can be calibrated to actual traffic data, but these two effects are not significantly affecting the results. Suzuki et al. [10] also represented driving behaviour of autonomous vehicles using IDM+ with different parameters representing human drivers. The second type of study reported the results of using Auto Cruise Controllers (ACC) to represent autonomous vehicles’ behaviour [11]. This type of research is more common in characterising travel behaviour than that of car-following models. The history of the ACC controllers is relatively new. Many ACC controllers have been developed based on simple structures, such as that of Wouter et al. [8] who used a Connected Auto Cruising Control (CACC) controller to investigate the possibility of dampening shock waves. Although various car-following models and ACC controllers have been used in many research works, there is not much evidence of a clear superiority of any of these models in terms of representation of autonomous vehicles. In the following section, work related to car-following models and ACC are reviewed and assessed in terms of their appropriateness for modelling AV.

### 2.1. Car-Following Models

Car-following models are microsimulation models and tools that are used to predict the longitudinal acceleration of a vehicle based on the speed and position of a leading vehicle in a traffic lane. The Helly model is a car-following model; the vehicle acceleration is controlled by the distance gap and speed difference with the following algorithm in Equation (1):

(1) $$\frac{{\mathrm{dv}}_{i}\left(t\right)}{\mathrm{dt}}=\alpha ∆v_{i}\left(t\right)+\beta \left(∆x_{i}\left(t\right)-\left(s_{0}+v_{i}\left(t\right)T\right)\right)$$

where:

*i* denotes the vehicle index
*v_i_*(*t*) is speed
Δ*v_i_*(*t*) is relative speed with respect to the preceding vehicle
Δ*x_i_*(*t*) is distance gap
*T* is desired time gap
*s*_0_ is the minimum gap at standstill
*α* is the sensitivity parameter with respect to relative speed Δ*v_i_*(*t*), and
*β* is the sensitivity parameter with respect to the difference between the current gap and the desired gap.

Moreover, the advanced version of this Helly model-type controller is formulated as follows (Equation (2)) [8,12]:

(2) $$\frac{{\mathrm{dv}}_{i}\left(t\right)}{\mathrm{dt}}=\min\left[k\left(v_{0}-v_{i}\left(t\right)\right),(\alpha ∆v_{i}\left(t\right)+\beta \left(∆x_{i}\left(t\right)-\left(s_{0}+v_{i}\left(t\right)T\right)\right)\right]$$

where *v*_0_ is desired speed and *k* is the sensitivity with respect to the difference between the desired speed and the current speed. 

The reason for adding the first formula is to distinguish between free driving behaviour and following behaviour. These Helly model-type controllers have no collision avoidance concept, so they are not able to represent braking behaviour adequately, such as emergency stopping. Other types of car-following models are available. These include for example the Intelligent Driver Model (IDM), IDM+ and Alexandros E. Papacharalampous’s Model (A.E.P.) which have been assessed in this study. Arne Kesting et al. [11] suggested that the IDM-type controller whose parameters are different from those of nonautonomous vehicles are best to use. The controller takes different parameters which include the desired time gap according to the traffic state such as ‘Free flow, ‘Congested traffic’, etc. The acceleration in this model is given in Equation (3) by:

(3) $$\frac{dv_{i}\left(t\right)}{dt}=\lambda_{a}a\left[\left(1-{\left(\frac{v_{i}\left(t\right)}{v_{0}}\right)}^{4}-{\left(\frac{s^{*}\left(v_{i}\left(t\right),∆v_{i}\left(t\right)\right)}{∆x_{i}\left(t\right)}\right)}^{2}\right)\right]\;s^{*}\left(v_{i}\left(t\right),∆v_{i}\left(t\right)\right)=s_{0}+v_{i}\left(t\right)\lambda_{T}T+\frac{v_{i}\left(t\right)∆v_{i}\left(t\right)}{2\sqrt{\lambda_{a}a\lambda_{b}b}}$$

where:

*a* is the maximum acceleration
*b* is the desired acceleration and
*λ_a_*, *λ_b_*, *λ_T_* are coefficients represent the multiplication factors

A feature of IDM [6] and IDM+ [7] is the nonlinear response to speed differences. These models are quite robust and user-friendly in simulating human driver behaviour. However, these models still have limitations in representing autonomous vehicle behaviour. Additionally, there is another type of controller that is based on the design of a flexible Model Predictive Controller, as presented in Equation (4) [13,14,15].

(4) $$\frac{dv_{i}\left(t\right)}{dt}=\left\{\begin{array}{c} k_{1}\left(v_{input,i}-v_{i}\left(t\right)\right)+k_{2}\frac{∆v_{i}\left(t\right)}{∆x_{i}\left(t\right)},\;\;\;\;\;\;\;\;\;if\;\;∆x_{i}\left(t\right)\leq \gamma^{ACC}\\ k_{1}\left(v_{0}-v_{i}\left(t\right)\right),\;\;\;\;\;\;\;\;\;if\;\;∆x_{i}\left(t\right)\geq \gamma^{ACC}\;\;\;\;\;\;\;\;\;\;\;\;\;\;\;\;\;\;\;\;\;\;\;\;\;\;\;\;\;\;\;\;\;\;\; \end{array}\right.\;v_{input,i}=min\left(\frac{∆x_{i}\left(t\right)-s_{0}}{T},v_{0}\right)$$

with

$∆x_{i}\left(t\right)>0\;\left(\mathrm{non}-\mathrm{collision}\;\mathrm{constraint}\right)$
$0\leq v_{i}\left(t\right)\leq v_{max}\;\left(\mathrm{physical}\;\mathrm{speed}\;\mathrm{range}\right)$
$a_{min}\leq \frac{dv_{i}\left(t\right)}{dt}\leq a_{max}\;\left(\mathrm{admissible}\;\mathrm{acceleration}\;\mathrm{range}\right)$

where:

variable *v_input,i_* is a gap-dependent desired speed
*γ^ACC^* is the onboard sensor detection range
*k*_1_ and *k*_2_ are feedback gains

A feature of this model is that the sensor detection range is clearly defined. This concept implies that the behaviour differs between within the sensor detection range and out of the range.

### 2.2. Appropriateness of Car-Following Models for Modelling AV

Table 1 below shows the most frequently used car-following models and their capabilities to replicate the behaviour of autonomous vehicles. There are three main requirements that need to be satisfied for these models to be useful in representing behaviour of autonomous vehicles. These are: the desired time gap, collision avoidance system and sensor detection range. Some models have limitations in representing varying desired gaps that depend on vehicle speed. For example, the GM model does not have the term of desired time gap or desired gap. Therefore, it cannot represent AV behaviour and it will have to be adjusted with the right parameters that depend on the speed. There are four car-following models and controllers that have a desired time-gap feature. These are Helly model, IDM, IDM+ and A.E.P. controller. Secondly, most autonomous vehicles are equipped with collision avoidance systems.

Therefore, controllers that are used to model AV should have such a function to represent collision avoidance behaviour. Any of these models could be considered for update in terms of collision avoidance systems in order to be able to model the control of autonomous vehicles. Finally, most car-following models do not have the concept of sensor detection range, only the ACC controller proposed by Alexandros E. Papacharalampous et al. [14] (hereinafter this is referred to as A.E.P. controller) has that concept. 

In conclusion, as mentioned above, various ACC controllers have been developed. The simplest controller is the Helly model-type controller, which has a classic structure and is sometimes used as the controller for Autonomous Vehicles (AV), but has no collision avoidance concept. Therefore, it is not capable of representing braking behaviour, such as emergency stopping. In this study, the Helly model is investigated for update to include a collision avoidance system module as discussed in the following Sections. 

## 3. Helly Model Investigation

In this Section, the Helly model will be investigated for its appropriateness for modelling autonomous vehicles. Two procedures that will be utilised in this paper to allow more realistic modelling of autonomous vehicles include: an examination of the model parameters, and then an update of the model to include the collision avoidance system. 

### 3.1. Assessment of Model’s Parameters

**Figure 1:**
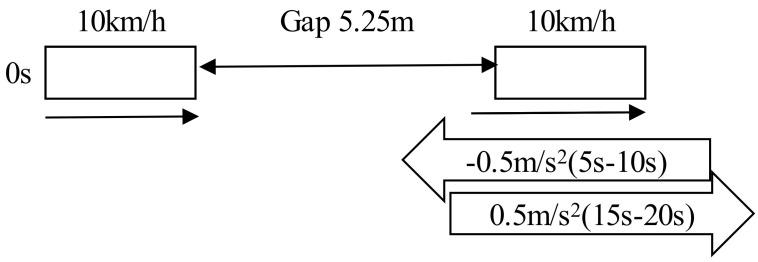
Representation of the following behaviour (example case).

**Figure 2:**
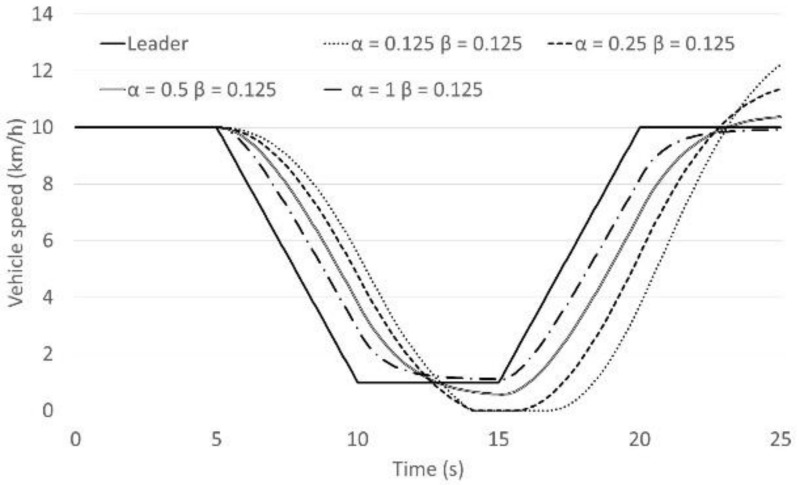
The following vehicle’s speed behaviour (*α* is fixed).

**Figure 3:**
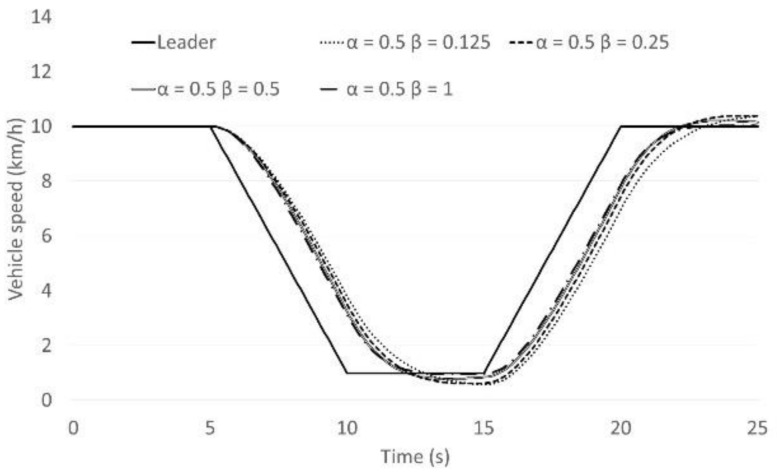
The following vehicle’s speed behaviour (*β* is fixed).

The Helly model is presented by Equation (1) above. There are two coefficients of the model; *α* which is the sensitivity parameter with respect to relative speed Δ*v_i_*(*t*), and *β* is the sensitivity parameter with respect to the difference between the current gap and the desired gap. Examining these coefficients is illustrated here by an example as presented in Figure 1. In this case, the leading vehicle and the following vehicle are both driving with a speed of 10 km/h and the first gap is 5.25 m. The leading vehicle is decelerating with a rate of 0.5 m/s^2^ from 5 s to 10 s, and then it accelerates with a rate of 0.5 m/s^2^ from 15 s to 20 s. The desired time gap was calculated using a very short setting of the time gap control strategy. The values of *α* and *β* are varied from 0.125 to 1, as illustrated in Figure 2 and Figure 3 below. 

Figure 2 and Figure 3 represent the following vehicle speed transition. From the results it appears that *α* has a much bigger impact on the car-following behaviour than *β*. Additionally, for values of *α* smaller than 0.5, such as 0.25 or 0.125, the following vehicle will not be able to follow the leading vehicle smoothly in congested flow. This tendency appears in the summarised parameter result of the Helly model [16]. Many researchers have tended to adjust the *β* coefficient but not the *α* coefficient. 

**Figure 4:**
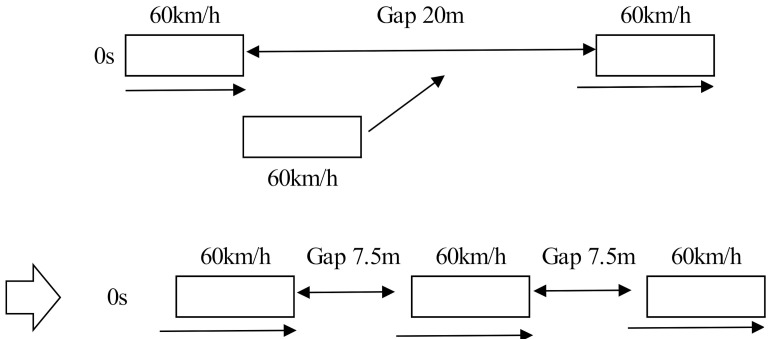
Representation of the cutting off behaviour (example case).

**Figure 5:**
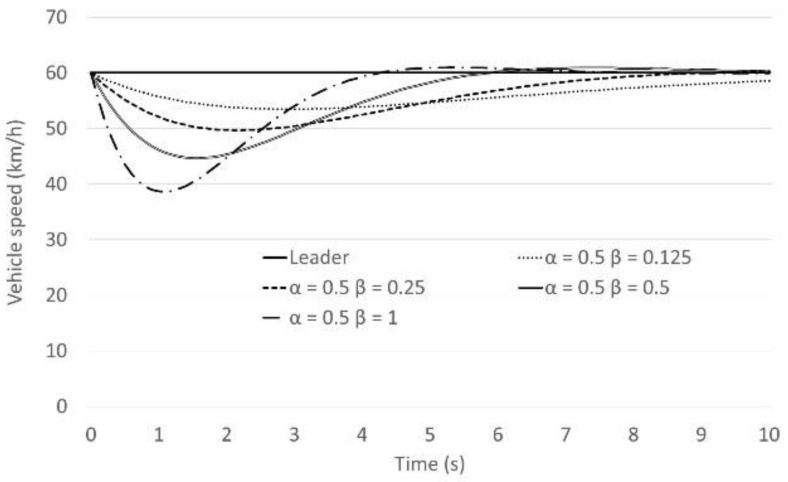
The following vehicle’s speed behaviour: Representation of the cutting off behaviour Speed (*α* is fixed).

**Figure 6:**
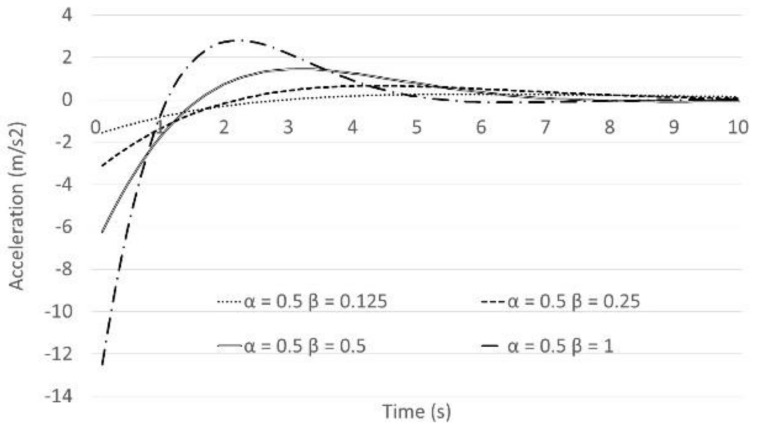
The following vehicle’s acceleration behaviour: Representation of the cutting off behaviour speed (*α* is fixed).

The next step is to assess the impact of the *β* coefficient on braking behaviour when another vehicle cuts in the gap. The example case is shown in Figure 4. The leading vehicle and following vehicle were driving at 60 km/h and the first gap was 20 m. Then, another vehicle cuts in the gap. The values of *β* were varied between 0.125 and 1 as shown in Figure 5 and Figure 6. Figure 5 shows the results of the following vehicle’s speed transition. Figure 6 shows the results of the following vehicle’s acceleration transition. According to the results, if the value of *β* is over 0.25, the following vehicle decelerates at a rate from 0.3 G to 1.3 G when another vehicle cuts in the gap. In other words, the following vehicle will need to make a fierce brake despite the fact that it is not actually a dangerous situation. That behaviour might cause rear-end collisions with the second or third following vehicles and results in unsafe and uncomfortable passenger experiences. In summary, in order to improve braking function of the Helly model, it seems that the original parameter values (*α* = 0.5 and *β* = 0.125) as were suggested by Helly, are best to use in the model. 

### 3.2. Modifying the Model Formulation

#### 3.2.1. Time-Gap Control Strategy

Most of the controllers for autonomous vehicles have been developed based on time-gap control policy and have the term of desired time gap *T* or *t_d_* in their formulas. While setting various desired time gaps is possible in reality, in many of the controllers, time gap is given as a constant value for simplicity. However, it is not very realistic to represent the behaviour of autonomous vehicles using a constant time gap. On the other hand, some of the controllers vary the desired time gap depending on the traffic conditions around the controlled vehicle [11,14]. Arne Ketsting et al. [11] proposed that the desired time gap should be halved in congested flows; therefore *λ_T_* was suggested by them to be set to 0.5. While this assumption might be effective in reducing congestion, it might not be possible to justify why all drivers have to opt to that choice. Some drivers who would not take a risky approach would possible rather choose to reduce the time gap values. If FACC is an extension of the traffic safety technology of lightening the burden of drivers, people expect that the desired time gap becomes bigger in congested flow because the distance gap is shorter than it is in free flow. Moreover, people would naturally expect that the desired time gap becomes smaller so as to prevent unnecessary cutting-in in free flow traffic. 

This study uses data from vehicles that are equipped with FACC and are already commercially available in the market, to assess the performance of car-following models and ACC controllers. Data on the standard gaps that have been obtained from 15 car manuals were attained and are presented in Table 2 below. Drivers who use FACC functions can select a setting for the desired gap range, such as long, middle or short according to the traffic situation as well as their own preferences. The number of settings and the length of distance gaps are not standardised among car manufactures. According to Toyota’s manual for example, the number of settings is three [17,18,19]. If a driver selects the middle setting, the autonomous vehicle will try to maintain a 40 m gap when the car drives at 80 km/h. Most of the manuals provide values of gaps at a vehicle high-speed range (80 km/h or 100 km/h). However, only Subaru’s manuals provide gap values at other values (40 km/h and 100 km/h). 

**Figure 7:**
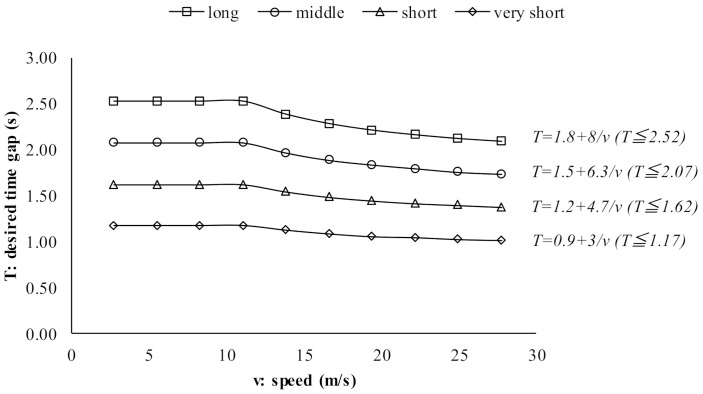
Time-gap control strategy.

Therefore, in this paper, time-gap control strategies to control autonomous vehicles are estimated based on Subaru’s manuals (gaps No.8 and No.9 in Table 2). From Table 2, it is clear that the time-gap values at 40 km/h are different to those values at 100 km/h. In terms of the desired gaps, car manuals state that when a vehicle stops, the distance gap will depend on the traffic condition regardless of the desired gap setting. Hence, when a vehicle stops, the desired gap was set at a value of 2 m regardless of the setting. The time-gap control strategy was estimated as presented in Figure 7. These values were estimated using the desired gaps at three levels of speed (0 km/h, 40 km/h and 100 km/h). These values are converted into the desired time gaps and then simply interpolated.

#### 3.2.2. The Updated Model

In this section we present the updated Helly model which is referred to here as Helly (FACC) model. In this case, the Helly model has been updated to deal with the collision avoidance-system problem. The concept of sensor detection range, the constraints of speed and acceleration and enhancing brake function were introduced into this model as presented in Equation (5) below. 

The Helly (FACC) model is formulated as follows:

(5) $$\frac{dv_{i}\left(t\right)}{dt}=\left\{\begin{array}{c} \delta \left(\alpha ∆v_{i}\left(t\right)+\beta \left(∆x_{i}\left(t\right)-\left(s_{0}+v_{i}\left(t\right)T\left(t\right)\right)\right)\right)\;\;if\;∆x_{i}\left(t\right)\leq s_{i}^{ACC}\\ \gamma \left(v_{0}-v_{i}\left(t\right)\right)\;\;\;\;\;\;\;\;\;\;\;\;\;\;\;\;\;\;\;\;\;\;\;\;\;\;\;\;\;\;\;\;\;\;\;\;\;\;\;\;\;\;\;\;\;\;\;\;\;\;\;\;\;\;\;\;if\;∆x_{i}\left(t\right)>s_{i}^{ACC} \end{array}\right.$$

(6) $$T\left(t\right)=\min\left(k_{1,\;setting}+k_{2,\;setting}/v_{i}\left(t\right),\;k_{3,\;setting}\right)$$

(7) $$\delta =\left\{\begin{array}{c} max\left(max\left(\frac{v_{i}^{2}}{2∆x_{i}b_{i}}-\frac{v_{i-1}^{2}}{2∆x_{i}b_{i-1}},\;0\right)+\frac{c}{∆x_{i}},1\right)\;\;\;\;\;if\;\;a<0\\ \;\;\;\;\;\;\;\;\;\;\;\;\;\;\;\;\;\;\;\;\;\;\;\;\;\;\;\;\;\;\;\;\;\;\;\;\;\;\;\;\;\;\;\;\;1\;\;\;\;\;\;\;\;\;\;\;\;\;\;\;\;\;\;\;\;\;\;\;\;\;\;\;\;\;\;\;\;\;\;\;\;\;\;\;\;\;if\;\;a\geq 0 \end{array}\right.\;$$

with

$0\leq v_{i}\left(t\right)\leq v_{max}\;$
$a_{min}\leq \frac{dv_{i}\left(t\right)}{dt}\leq a_{max}\;$

where:

*α* is the sensitivity parameter with respect to relative speed Δ*v_i_*(*t*), and equals 0.5,
*β* is the sensitivity parameter with respect to the difference between the current gap and the desired gap and equals 0.125,
*γ* is the onboard sensor detection range and equals 0.2,
$s_{i}^{ACC}$ is the minimum gap at standstill and equals 120 m,
*a_min_* is minimum acceleration and equals −8 m/s^2^ and
*a_max_* is maximum acceleration and equals 0.6 m/s^2^,
*δ* is defined as the safety risk

The values of the parameters that are related to the time-gap control strategy, $k_{1,\;setting}$, $k_{2,\;setting}$ and $k_{3,\;setting}$, are given in Table 3. 

**Figure 8:**
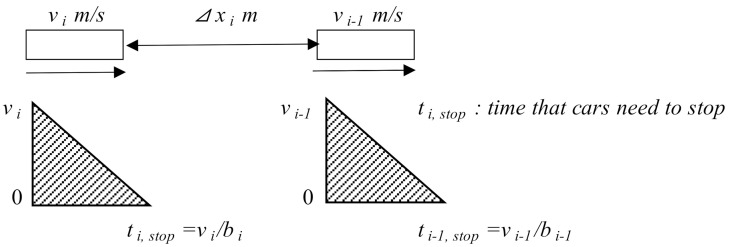
Representation of concept of safety risk.

In the above model, Equation (5) controls the acceleration of the vehicle, Equation (6) represents the time gap control strategy. These equations are the same equations as in the original Helly model. Equation (7) is added to account for the collision avoidance system in an attempt to enhance the brake function of the model. In this case, the *δ* parameter is defined as the safety risk, which is introduced in this model as a function of gap settings, *v_i_*, the speed and Δ*x_i_*(*t*) is the distance gap, *b_i_* and *b_i_*_−1_ are decelerations equal to 0.3 G (2.97 m/s^2^) and *c* is 4 m. This concept of the safety risk is based on the Mazda algorithm [17,18], and is illustrated in Equations (8) and (9) and in Figure 8. 

This function works especially well when a vehicle is in dangerous situation (e.g., emergency stop). On the other hand, the function does not work when a vehicle cuts into the short gap in front of the controlled vehicle. In this case, in order for the vehicle to stop safely, we have:

(8) $$\frac{v_{i-1}t_{i-1,\;\mathrm{stop}}}{2}+∆x_{i}>\frac{v_{i}t_{i,\;\mathrm{stop}}}{2}+c$$

Which leads to:

(9) $$1>\frac{1}{∆x_{i}}\left(\frac{v_{i}^{2}}{2b_{i}}-\frac{v_{i-1}^{2}}{2b_{i-1}}+c\;\right)$$

where:

*t_i_*, time that vehicle needs to stop
*b_i_*, *b_i_*_−1_ are decelerations (m/s^2^) and
*c* is the desired safe distance (m)

#### 3.2.3. Model Validation

To validate the updated model, the braking behaviour of five models, the Helly model, Helly (FACC) model, IDM, IDM+ and A.E.P. controllers have been assessed and compared. The purpose in this case is to investigate how the models perform in terms of meeting the standards of the collision avoidance systems. Data from the Japanese collision avoidance system standards have been utilised in order to set some reference values for the collision avoidance system. The Japanese government publishes standards of collision avoidance systems. These standards have three sets of paradigms; the first paradigm states that “when the following vehicle approaches at 50 km/h to a stationary vehicle, the following vehicle should not collide with the stationary vehicle or the following vehicle’s speed should be less than 20 km/h if a collision occurs”. The second paradigm states that “when the following vehicle approaches at 50 km/h to a leading vehicle that is driving at 20 km/h, no collision should occur”. The third paradigm states that “an alarm to inform drivers of the existence of a vehicle which the vehicle may collide with has to set at least 0.8 s before the collision avoidance system works”. 

In this Section, the braking behaviour of the five models (Helly model, Helly (FACC), IDM, IDM+ and A.E.P. controller) are assessed and compared using the first and second sets of the Japanese standards of collision avoidance systems, as presented in Table 4. In this case, the parameter values of the Helly model, *α* and *β*, are assumed to be the same as those of the updated Helly (FACC) model which are 0.5 and 0.125. The parameter values of IDM and IDM+ are assumed to be *a* = 0.6, *b* = 2.8 and *v*_0_ = 60 km/h, based on the work carried out by Suzuki et al. [10] representing the current autonomous vehicle’s performance. The parameter values of the A.E.P. controller are assumed to be: *γ^ACC^* = 120 m, *k*_1_ = 0.2 and *k*_2_ = 15 as reported by [20,21]. 

**Figure 9:**
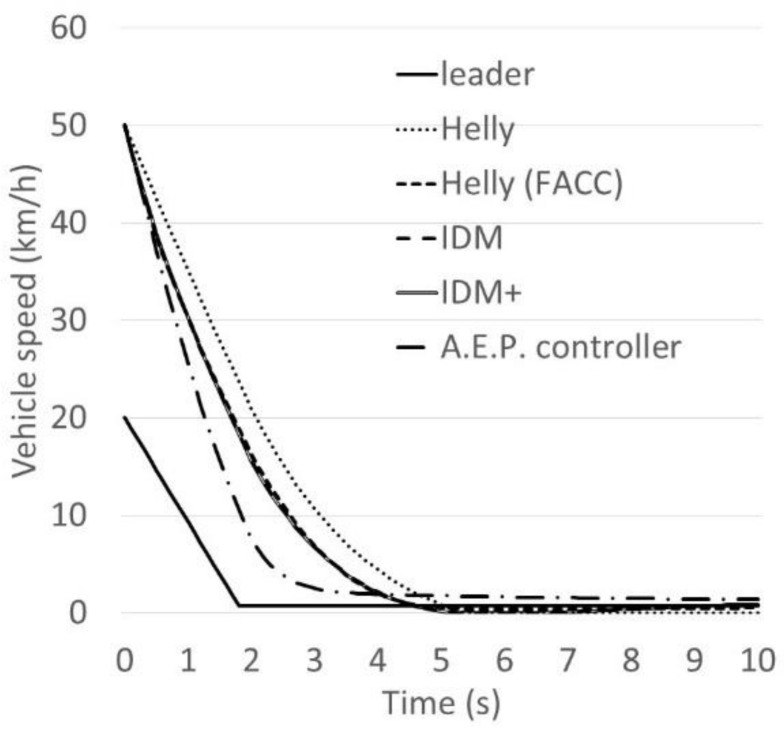
The following vehicle’s speed transition of case No.1.

**Figure 10:**
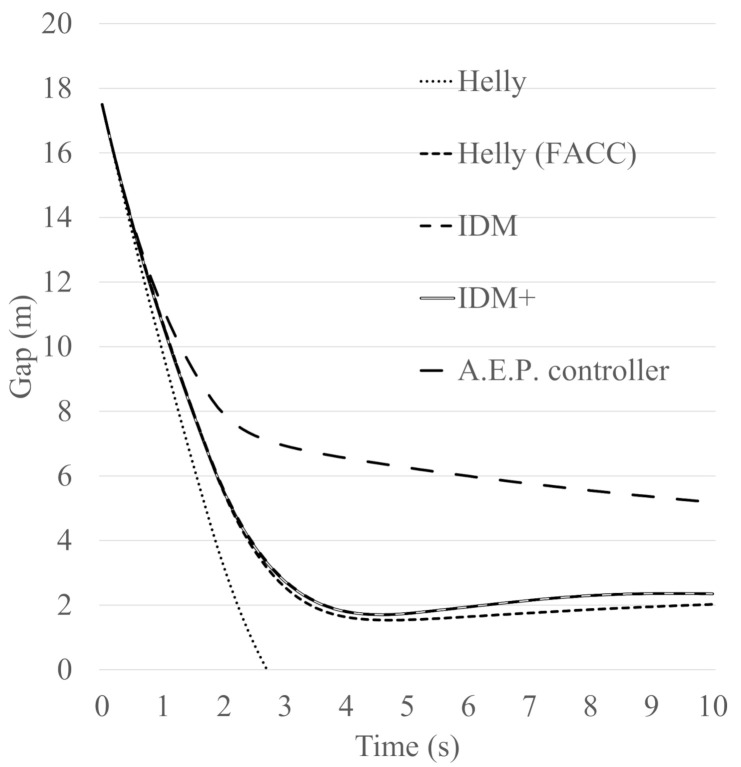
The following vehicle’s gap transition of case No.1.

**Figure 11:**
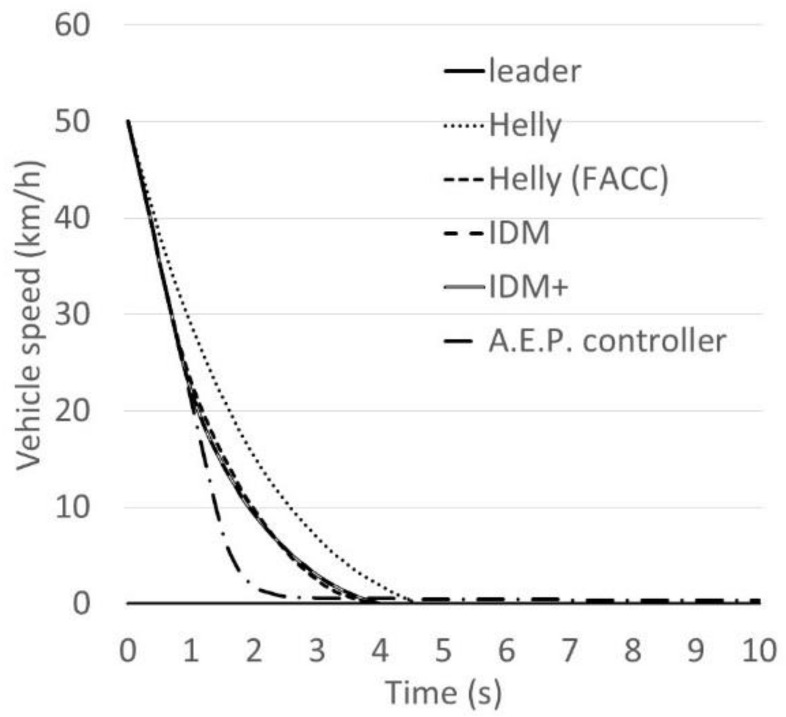
The following vehicle’s speed transition of case No.2.

**Figure 12:**
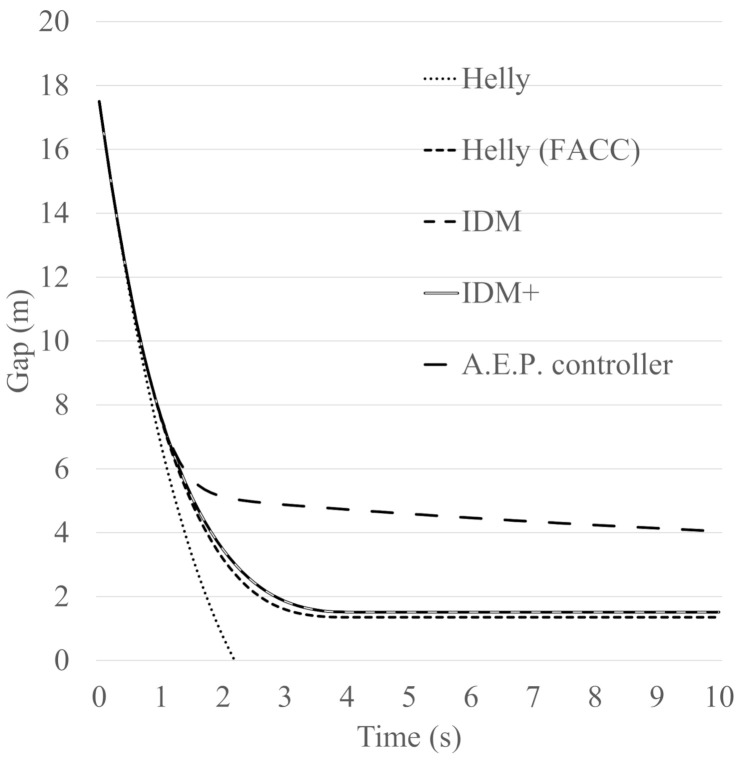
The following vehicle’s gap transition of case No.2.

The first gap that was calculated using the desired time gap at speed of 50 km/h is 17.5 m. Figure 9 and Figure 10 show the following vehicle speed and gap transition of case No.1, while Figure 11 and Figure 12 show the following vehicle speed and gap transition of case No.2. From the results, the four models and controller meet the standards of collision avoidance systems. Only the Helly model does not; it shows a collision rather than maintaining a gap similar to the other models (Figure 10 and Figure 12 for both cases). This is because the Helly model does not have a collision avoidance function in it. 

The modified model performs better. The braking ability of the Helly (FACC) model is comparable to that of IDM or IDM+. It should be noted here, that the A.E.P. controller shows very good performance at reducing speed in an emergency. These results are very valuable not only for traffic modelling and accurate prediction of AV behaviour, but also for environmental benefits since there will be benefits in controlling the speed, acceleration, deceleration and savings regarding fuel consumption [22,23,24,25,26].

## 4. Conclusions and Further Research Recommendations

The study reports on an investigation of the appropriateness of car-following models in replicating the behaviour of autonomous vehicles. Data from vehicles that are equipped with full-range ACC (FACC) and are already commercially available in the market were obtained and utilised in the assessment. The Helly model, which is a very frequently used car-following model, is assessed and examined for appropriateness for update for the purpose of using it to model autonomous vehicles more efficiently. This involved assessing the parameters of the model and investigating a possible update to include a collision avoidance-system component. 

An enhanced car-following model, Helly (FACC), is proposed, assessed and validated. The validation included a comparative analysis of the models’ performance in two paradigms that are taken from the Japanese collision avoidance-system standards. The two cases are: a paradigm that states that “when the following vehicle approaches at 50 km/h to a stationary vehicle, the following vehicle should not collide with the stationary vehicle or the following vehicle’s speed should be less than 20 km/h if a collision occurs”. The second paradigm states that “when the following vehicle approaches at 50 km/h to a leading vehicle that is driving at 20 km/h, no collision should occur”. The braking behaviour of five models, the Helly model, Helly (FACC) model, Intelligent Driver Model (IDM), IDM+ and Alexandros E. Papacharalampous’s Model (A.E.P.) controllers have been compared and analysed. 

The results have been assessed in terms of the following vehicle’s speed and gap transition of the two investigated standards of collision avoidance systems. From the results, it appears that four out of the five investigated models and controller meet the standards of collision avoidance systems. One model did not perform as well which is the original Helly model. In this case, the Helly model shows a collision rather than maintaining a safe gap between the leader vehicle and the following vehicle. This is because the Helly model does not have a collision avoidance function within it. 

The modified model performs better as a result of having a braking function. The performance of the Helly (FACC) model is found to be comparable to that of IDM or IDM+. The results also show that the A.E.P. controller performs significantly better than the other models. These results are very valuable not only for traffic modelling and accurate prediction of AV behaviour, but also for the environment, since there will be benefits to controlling the speed, acceleration and deceleration, and savings in fuel consumptions.

There has been intense progress in ITS, vehicle and information technologies over the past few decades. Many research studies have been undertaken to investigate impacts of such technologies on driving and traffic behaviour. However, the full impact of this technology is yet to be discovered. This is because the technology is not widely available yet and further information is needed for full realisation of its effects. Most of the studies are implemented using simulations or surveys, rather than monitoring actual behaviour. Social and technological issues and predictions of the market have to be further investigated and documented. Further research is typically needed in all these areas. Further areas that are also suggested for future research include the impacts of using technological solutions and autodriving on fuel saving and environmental benefits. In order to enumerate the extent of the validity of the results and representativity to real data, future work should also attempt to compare the results obtained from simulations with more real-world data. AV are becoming a reality, and very soon, data on these types of vehicles will become more available. Therefore, there will be more chances for further, interesting research.

## Figures and Tables

**Table 1:** The main features of autonomous vehicles that need to be represented in models.

Car-Following Model or ACC Controller	Desired Time Gap or Desired Gap	Collision Avoidance System	Sensor Detection Range
GM model	X	X	X
Collision Avoidace (CA) model	X	X	X
Helly model	✓	X	X
Optimal Velocity Model (OVM)	X	X	X
Intelligent Driver Model (IDM)	✓	X	X
Intelligent Driver Model (IDM)+	✓	X	X
Controller proposed by A. E. Papacharalampous	✓	X	✓

✓: The feature is represented in model. X: The feature is not represented in model.

**Table 2:** Desired gap of FACC function.

Car Manufacturer.		Toyota	Honda		Nissan		Mazda		Subaru			
Gap No.		1	2	3	4	5	6	7	8	9	10	11
Gap (m)	very long		61	78								
	long	50	47	59	60	60	50	50	30	60	30	60
	middle	40	33	40	45	50	40	40	25	50	22	45
	short	30	25	30	30	40	30	30	20	40	15	30
	very short						25	20	15	30		
Speed		80 km/h	80 km/h	100 km/h	100 km/h	100 km/h	80 km/h	80 km/h	40 km/h	100 km/h	40 km/h	100 km/h
vehicle type		Alphard (2018.3) crown majesta (2017.5) prius (2018.3)	legend (2018)		Cima (2017.6) fuga (2017.11)	elgrand (2015.4)	cx-8 (2018.3) cx-5 (2018.2)	atenza (2017.8)	legacy (2017.10) impreza (2017.10) xv (2017.5)		levorg (2017.8) forester (2017.4)	

**Table 3:** Values of $k_{1,\mathrm{setting}}$, $k_{2,\mathrm{setting}}$ and $k_{3,\mathrm{setting}}$.

Setting	*K*_1,_ *_setting_*	*K*_2,_ *_setting_*	*K*_3,_ *_setting_*
Very short	1.8	8.0	2.52
Short	1.5	6.3	2.07
Middle	1.2	4.7	1.62
Long	0.9	3.0	1.17

**Table 4:** Validation of collision avoidance system.

Standard	Situation	Time Gap Setting	Follower (km/h)	Leader (km/h)	First Gap (m)	Behaviour of Follower
No.1	Emergency stop	Very short	50	0	17.5	Stop
No.2	Emergency stop	Very short	50	20	17.5	Decelerate (−2.97 m/s^2^ (0.3 G)) from 0 s–1.8 s

## Data Availability

Not applicable.

## Nomenclature

AV	Autonomous Vehicles
A.E.P Model	Alexandros E. Papacharalampous’s Model
CACC	Connected Auto Cruising Control
FACC	Full-range Auto Cruising Control
GM	Gipps Model
Helly (FACC)	Enhanced Helly car-following model
IDM	Intelligent Driver Model
IDM+	Intelligent Driver Model+
ISA	Intelligent Speed Adaptation
ITS	Intelligent Transportation Systems
OVM	Optimal Velocity Model
